# Transepithelial corneal cross-linking assisted by two continuous cycles of iontophoresis for progressive keratoconus in adults: retrospective 5-year analysis

**DOI:** 10.1007/s00417-020-04861-y

**Published:** 2020-07-29

**Authors:** Huping Wu, Shunrong Luo, Xie Fang, Xumin Shang, Zhiwen Xie, Xianwen Xiao, Zhirong Lin, Zuguo Liu

**Affiliations:** 1 Eye Institute and Affiliated Xiamen Eye Center of Xiamen University, Xiamen, China; 2 Fujian Provincial Key Laboratory of Ophthalmology and Visual Science, Xiamen, China; 3 Fujian Provincial Key Laboratory of Corneal & Ocular Surface Diseases, Xiamen, China

**Keywords:** Progressive keratoconus, Iontophoresis, Transepithelial corneal crosslinking, Tomography, In vivo confocal scanning microscopy

## Abstract

**Purpose:**

The aim of this study is to compare the long-term effects of transepithelial corneal crosslinking with two continuous cycles of iontophoresis (EI-CXL) and conventional corneal crosslinking (C-CXL) in adults with progressive keratoconus.

**Methods:**

A retrospective analysis was conducted in adults who underwent C-CXL or EI-CXL between 2013 and 2015. Visual acuity, corneal tomography, anterior segment optical coherence tomography, in vivo corneal confocal microscopy (IVCM), and endothelial cell count (ECC) were performed preoperatively and 5 years postoperatively.

**Results:**

Sixty-eight patients with a mean age of (24.3 ± 3.8) years were included, 34 for each group. After CXL, UCVA or BCVA remained stable, while the spherical diopter, cylinder diopter, spherical equivalent, and *K*_max_ significantly decreased at 1, 2, and 3 years in both groups than baseline (*P* < 0.05). No significant differences were found in any refractive or tomographic parameters as well as the minimal corneal thickness between groups during follow-up. At 5 years, *K*_max_ was slightly higher in EI-CXL group (58.16 ± 6.28) than that of C-CXL group (57.46 ± 4.98). At 3 and 5 years, the minimal corneal thickness in C-CXL group was still significantly lower than baseline (*P* < 0.05). IVCM demonstrated the demarcation zone at a mean depth of (302.0 ± 41.7) μm after C-CXL, and at (251.2 ± 28.1) μm after EI-CXL (*P* < 0.001). Keratocyte repopulation was detectable at all follow-up timepoint in both groups. Postoperative complications including progression were recorded in 6 patients (11.7%) after C-CXL and 3 patients (8.8%) after EI-CXL. ECC remained stable in both groups.

**Conclusion:**

EI-CXL showed approximate efficacy with C-CXL in stabilizing progressive keratoconus in adults. EI-CXL has the potential to be a preferable transepithelial protocol.

## Introduction

Keratoconus (KC) has long been considered as a progressive, non-inflammatory corneal thinning and ectasia with reduced biomechanical stability, which may lead to severe visual impairment in young and even pediatric patients. In very severe cases, lamellar or penetrating corneal transplantation is the final therapeutic option to regain vision. In recent years, corneal collagen crosslinking (CXL) has been recognized as a safe and effective treatment to delay or halt further progression of KC and can reduce the need of keratoplasty [1]. During CXL, riboflavin interacts with ultraviolet-A light to create crosslinking of protein fibrils followed by formation of interchain disulfide bonds, thus arresting the progression of corneal ectasia by increasing the biomechanical stability of the cornea. CXL has been considered as one of the standard treatments of progressive keratoconus worldwide.

Various protocols [1] of CXL have been extensively investigated and applicated. CXL using classic Dresden’s protocol with epithelium removal (conventional CXL) showed long-term efficacy of stabilization and improvement for KC. However, postoperative complications using standard epi-off protocol [2–5], such as corneal haze, sterile corneal infiltrates, recurrent erosion syndrome, have been reported and should be taken into consideration. To reduce the risk of postoperative complications, epithelium-on(epi-on) protocols assisted by iontophoretic delivery or transepithelial riboflavin were brought into sight and were considered by some researchers to be a better choice.

Iontophoresis, in which an electrical gradient is used to drive negatively charged riboflavin molecules across the intact epithelium, may further enhance riboflavin penetration in transepithelial CXL. Laboratory and clinical studies [6–9] of iontophoresis have been encouraging, demonstrating increased transepithelial penetration of riboflavin and improvement of corneal biomechanics. Nevertheless, most studies showed inferior results of standard protocol of iontophoresis when compared to epi-off protocol [10–13]. The limited depth of riboflavin penetration and lower concentration of riboflavin in the corneal stroma were the critical inadequacies of standard iontophoresis protocol [14]. To overcome this shortcoming, transepithelial CXL assisted by two continuous cycles of iontophoresis [15] was taken into consideration, and some study showed better short-term outcome than that by standard iontophoresis [15]. Theoretically, two continuous cycles of standard iontophoresis (enhance iontophoresis) allowed time for riboflavin to penetrate and diffuse more posteriorly. However, the long-term effect of this modified iontophoretic protocol remained unclear.

This study aimed to compare the long-term efficacy of enhanced iontophoresis-assisted transepithelial corneal crosslinking (EI-CXL) and conventional corneal crosslinking (C-CXL) in adults with progressive keratoconus, as well as the characteristics in visual acuity, corneal topography, and morphological alteration.

## Methods

### Patients and criteria

This retrospective nonrandomized study comprised the patients who were diagnosed with progressive keratoconus and underwent a C-CXL or EI-CXL procedure between January 2013 and January 2015 at the affiliated Xiamen Eye Center of Xiamen University, China. The diagnosis of keratoconus was established in concordance with the consensus of keratoconus and ectatic corneal diseases [16]. The following features were defined to be the inclusion criteria for the study: (1) diagnosed as patients as progressive keratoconus and aged ≥ 14 years; (2) at least 60 months of follow-up postoperatively. For patients with bilateral progressive keratoconus, only the right eye was included. Exclusion criteria were (1) patients with a minimal corneal thickness lower than 400 μm, (2) patients aged < 14 years, (3) patients with maximum keratometry (*K*_max_) higher than 60.0 diopter (D), and (4) patients with irregular or incomplete follow-up. Written informed consent was obtained from patients themselves. The study and surgical protocol were both approved by the hospital’s ethics committee and were performed according to the tenets of the Declaration of Helsinki.

Before diagnosed as progressive keratoconus [17–20], patients were followed up for at least 12 months. Besides an increase in *K*_max_ of ≥ 1.0 D, progressive keratoconus was defined with any of the following criteria that occurred during the 12 months: (1) a 5% or more reduction in thickness of the thinnest point of the cornea obtained by corneal tomography; (2) an increase in cylindrical value of ≥ 1.0 D, or in spherical equivalent ≥ 0.5 D; and (3) loss of at least 2 lines of vision in BCVA within 12 months.

### Surgical procedures of C-CXL and EI-CXL

Before surgery, topical 0.1% pilocarpine eye drops were instilled 30 min before surgery. Topical 0.5% proparacaine hydrochloride eye drops were instilled twice before surgery (every 5 min). All surgical procedures were performed in a sterile operating room.

For C-CXL, the central 9.0 mm (diameter) corneal epithelium was removed by mechanical debridement using a blunt spatula. After epithelial abrasion, 0.1% solution of riboflavin in 20% dextran (Ricrolin, SOOFT, Italy) was applied to the cornea every 1 min for 20 min. The central cornea was then irradiated (UVX-2000, IROC, Switzerland) with a light spot of 9 mm diameter for 10 min at a 9 mW/cm^2^UV-A light (5.4 J/cm^2^ surface dose). Riboflavin solution was further applied every 2 min during the UV-A irradiation. At the end of surgery, a soft bandage contact lens was placed for 1 week.

For EI-CXL, the return electrode was affixed to the skin of frontal region, while the corneal iontophoresis electrode was attached to the cornea by a vacuum adsorption device (SOOFT, Italy). The corneal electrode was filled with approximately 0.5 mL of 0.1% riboflavin solution (Ricrolin^+^, SOOFT, Italy), which was specifically designed for iontophoretic delivery of riboflavin, from the open proximal side until the stainless steel mesh was completely immersed. After that, the device was connected to a constant current generator (I-ON XL, SOOFT, Italy) set at 1 mA current. The total dose of 10 mA/10 min (continuous two cycles of standard iontophoretic delivery) was monitored by the generator. After completion of iontophoresis, the UV light was then focus on the apex of the cornea through the four-spot alignment system. The cornea was irradiated at the same dose of UV-A as C-CXL. During irradiation, drops of balanced solution were applied to the cornea every 1 min to keep moisture and rinse away residual riboflavin. No soft contact lens was placed after surgery.

For both protocols, tobramycin and dexamethasone eye ointment (Alcon, Novartis, Switzerland) was applied to the conjunctival sac postoperatively. Subsequent treatment included 0.5% loteprednol and tobramycin eye drops four times per day and tapered over 4 weeks, topical artificial tears of 0.3% hyaluronate sodium four times per day for at least 8 weeks. Patients were investigated before surgery and at 1, 2, 3, and 5 years after CXL treatment as follows.

### Postoperative follow-up

In the preoperative and postoperative examinations, the following parameters were accessed: uncorrected distance visual acuity (UCVA), best corrected distance visual acuity (BCVA), slit-lamp microscopy examination including corneal fluorescein sodium staining (BQ900IM9900, Haag-Streit, Switzerland), corneal tomography and pachymetry (Pentacam HR 70900, Oculus, Wetzlar, Germany), anterior segment optical coherence tomography (AS-OCT, Visante OCT, Carl Zeiss Meditec Inc., Germany), endothelial biomicroscope (SP-3000P, Topcon, Tokyo, Japan), and in vivo corneal confocal microscopy (IVCM, HRT3/Rostock Cornea Module, Heidelberg Engineering GmbH, Germany). *K* values (*K*_max_, K1 and K2) and minimum pachymetry values were derived from the tomography data. All patients were assessed at baseline and followed up for 5 years postoperatively. The UCVA and BCVA were converted to logarithm of the minimum angle of resolution (logMAR) units for statistical analysis.

### Statistical analysis

The data was imported to the Statistical Package for Social Sciences (SPSS Inc., Chicago, IL, version 16.0) for analysis. Two-way repeated measures ANOVA and student’s *t* test were used for statistical comparisons as appropriate. Bonferroni correction was made for multiple comparisons. For binary outcomes, the stratified Cochran chi-square test was used for comparisons of proportions between groups. The significance level was set at < 0.05.

## Results

### Demographics of patients

A total of 133 patients with keratoconus underwent C-CXL or EI-CXL in the affiliated Xiamen Eye Center of Xiamen University between January 2013 and January 2015. In these patients, 65 treated cases were excluded in the study by reasons of age less than 14 years, minimal corneal thickness lower than 400 μm, *K*_max_ higher than 60.0 D, and rare or irregular follow-up. Eventually, a total of 68 patients (68 eyes) were included, 34 patients for each group. Patient demographics are listed in Table 1. The baseline values of the two groups were comparable, including age, sex ratio, ratio of right eyes, and ratio of history with allergic conjunctivitis (all *P* > 0.05).

**Table 1:** Patients demographics for all subjects included in this study

	C-CXL group (*n* = 34)	EI-CXL group (*n* = 34)	All patients, (*n* = 68)	*P* value
Age (years)	24.9 ± 4.0	23.8 ± 3.6	24.3 ± 3.8	0.236
Sex ratio (M/F)	20/14	16/18	36/30	0.331
Side (OD/OS)	15/19	16/18	31/37	0.808
*K*_max_	58.37 ± 4.96	58.26 ± 5.78	58.31 ± 5.34	0.917
AC (Y/N)	25/9	23/11	48/20	0.595

*M*/*F* male/female, *AC* allergic conjunctivitis; data was presented with mean ± standard deviation

### Refractive and tomographic changes after C-CXL and EI-CXL

Comparative analysis of the UCVA and BCVA as well as refractive parameters at all follow-up period was shown in Table 2. After CXL, UCVA or BCVA remained stable in each group, and no statistical difference was found between the two groups. After CXL, the spherical, cylinder diopter as well as the spherical equivalent value significantly decreased at 1, 2, and 3 years in both group when compared with baseline (*P* < 0.05). Although the refractive data of spherical, cylinder diopter, and spherical equivalent showed slightly higher absolute value in the EI-CXL group at 5 years, no significant difference was found between the two groups at any follow-up time.

**Table 2:** Refractive and tomographic changes after transepithelial CXL assisted by enhanced iontophoresis (*n* = 34 for each group)

	Group	Baseline	1 year		2 years		3 years		5 years	
		Mean ± SD	Mean ± SD	*p*	Mean ± SD	*p*	Mean ± SD	*p*	Mean ± SD	*p*
UCVA	c-CXL	0.94 ± 0.29	0.94 ± 0.30	1.000	0.99 ± 0.34	0.428	0.96 ± 0.32	1.000	0.99 ± 0.33	0.378
	ei-CXL	0.97 ± 0.33	0.97 ± 0.34	1.000	1.01 ± 0.36	0.424	1.00 ± 0.35	0.703	1.02 ± 0.33	1.000
	*p*	0.371	0.424		0.388		0.252		0.546	
BCVA	c-CXL	0.30 ± 0.08	0.30 ± 0.11	1.000	0.29 ± 0.11	1.000	0.31 ± 0.11	1.000	0.31 ± 0.08	1.000
	ei-CXL	0.30 ± 0.11	0.29 ± 0.99	1.000	0.29 ± 0.12	1.000	0.33 ± 0.12	1.000	0.33 ± 0.11	1.000
	*p*	0.898	0.789		0.902		0.528		0.581	
Sphere, D	c-CXL	− 3.76 ± 2.25	− 3.15 ± 1.73	0.005	− 3.29 ± 1.82	0.002	− 3.32 ± 1.92	0.031	− 3.41 ± 1.90	0.062
	ei-CXL	− 3.76 ± 1.46	− 3.51 ± 1.32	0.029	− 3.54 ± 1.29	0.001	− 3.56 ± 1.25	0.016	− 3.71 ± 1.54	1.000
	*p*	0.984	0.256		0.416		0.430		0.328	
Cylinder, D	c-CXL	− 3.87 ± 1.67	− 3.16 ± 1.46	0.005	− 3.13 ± 1.41	0.002	− 3.24 ± 1.39	0.035	− 3.28 ± 1.44	0.071
	ei-CXL	− 3.88 ± 1.91	− 3.21 ± 1.35	0.000	− 3.31 ± 1.43	0.000	− 3.52 ± 1.63	0.003	− 3.76 ± 1.94	1.000
	*p*	0.986	0.883		0.626		0.470		0.270	
SE, D	c-CXL	− 5.70 ± 2.50	− 4.73 ± 1.88	0.001	− 4.86 ± 1.94	0.001	− 4.95 ± 2.09	0.005	− 5.05 ± 2.07	0.022
	ei-CXL	− 5.70 ± 1.67	− 5.12 ± 1.41	0.000	− 5.19 ± 1.43	0.000	− 5.32 ± 1.51	0.000	− 5.59 ± 1.84	1.000
	*p*	0.994	0.309		0.352		0.300		0.154	
K1, D	c-CXL	48.27 ± 4.24	48.05 ± 4.20	0.060	48.15 ± 4.23	1.000	48.26 ± 4.45	1.000	48.29 ± 4.43	1.000
	ei-CXL	48.49 ± 5.21	48.30 ± 5.22	0.002	48.38 ± 5.28	0.076	48.48 ± 5.33	1.000	48.63 ± 5.51	1.000
	*p*	0.819	0.799		0.819		0.832		0.744	
K2, D	c-CXL	52.68 ± 3.85	52.09 ± 3.81	< 0.001	52.14 ± 3.83	0.003	52.27 ± 3.99	0.177	52.32 ± 3.99	0.363
	ei-CXL	52.65 ± 5.38	52.09 ± 5.20	< 0.001	52.22 ± 5.08	0.001	52.37 ± 5.29	0.135	52.88 ± 5.78	1.000
	*p*	0.979	0.995		0.927		0.912		0.573	
*K*_max_, D	c-CXL	58.37 ± 4.96	56.81 ± 4.04	0.020	56.78 ± 4.02	0.001	57.05 ± 4.82	0.048	57.46 ± 4.98	0.681
	ei-CXL	58.26 ± 5.78	56.94 ± 5.42	< 0.001	57.06 ± 5.60	< 0.001	57.30 ± 5.77	< 0.001	58.16 ± 6.28	1.000
	*p*	0.917	0.883		0.755		0.798		0.523	
Minimal thickness, μm	c-CXL	430.8 ± 30.5	412.0 ± 31.2	< 0.001	421.5 ± 30.9	< 0.001	426.6 ± 31.6	0.006	427.2 ± 31.4	0.036
	ei-CXL	430.4 ± 28.6	416.5 ± 30.0	< 0.001	425.9 ± 27.9	< 0.001	429.7 ± 28.4	1.000	430.5 ± 29.6	1.000
	*p*	0.953	0.550		0.554		0.682		0.666	
EC, cells/mm^2^	c-CXL	2666 ± 256	2650 ± 266	1.000	2654 ± 294	1.000	2628 ± 349	1.000	2604 ± 357	1.000
	ei-CXL	2697 ± 244	2688 ± 247	1.000	2681 ± 276	1.000	2634 ± 279	1.000	2664 ± 306	1.000
	*p*	0.611	0.488		0.677		0.934		0.420	
IOP, mmHg	c-CXL	14.53 ± 2.00	14.59 ± 2.40	1.000	14.65 ± 2.84	1.000	14.62 ± 2.64	1.000	14.53 ± 2.50	1.000
	ei-CXL	14.56 ± 1.76	14.56 ± 3.08	1.000	14.50 ± 2.55	1.000	14.38 ± 2.81	1.000	14.71 ± 2.17	1.000
	*p*	0.955	0.963		0.837		0.756		0.767	

*UCVA* uncorrected visual acuity, *BCVA* best-corrected visual acuity, *SE* spherical equivalent, *EC* endothelial cell, *IOP* intraocular ocular pressure

After CXL, corneal flattening was seen with significant decreases in *K*_max_ (at 1, 2, and 3 years, *P* < 0.05) and K2 (at 1 and 2 years, *P* < 0.01) in each group when compared with the baseline; however, the *K*_max_ and K2 values at 5 years after surgery were comparable to baseline (*P* > 0.05). No significant difference in K1, K2, or *K*_max_ was found between the two groups at any follow-up time (*P* > 0.05). The corneal thickness of the thinnest point significantly reduced at all the follow-up time after C-CXL (*P* < 0.05), but reduced only at 1 and 2 years after EI-CXL (*P* < 0.001). Likewise, no difference was found in corneal thickness of the thinnest point between the two groups at any follow-up time (*P* > 0.05).

### Structural alteration in the corneal stroma

**Fig. 1:**
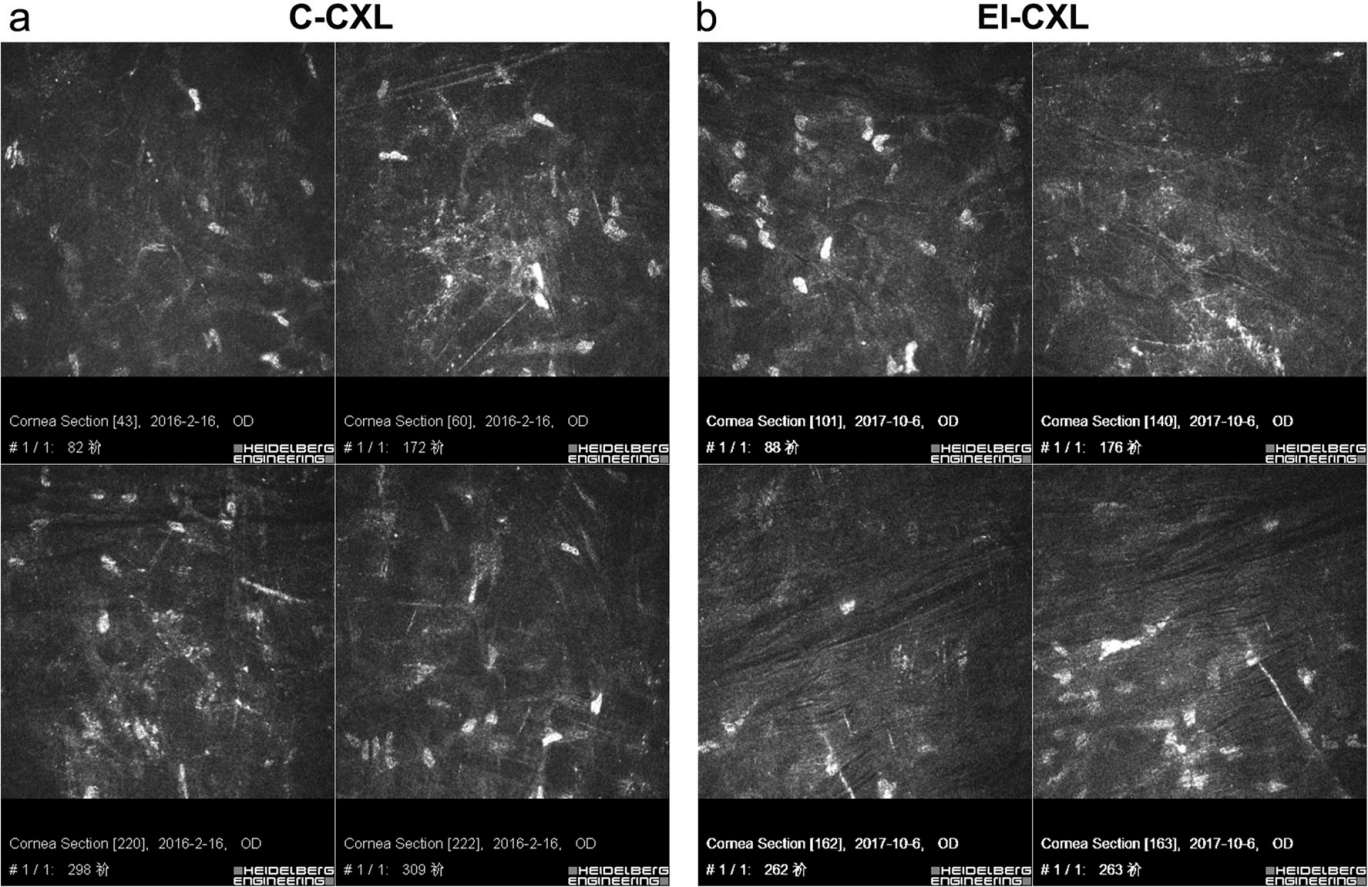
In vivo confocal microscopy scans of the corneal stroma at 3 years after C-CXL (patient a) and EI-CXL (patient b). In patient a, consecutive scans at different corneal depth: 82, 172, 298, and 309 μm. In patient b, consecutive scans at similar corneal depth: 88, 176, 262, and 263 μm. The transition zone from acellular to cellular corneal stroma was at 298 and 262 μm in patient a and b, respectively

At 1 month after CXL, demarcation line was visible in a low proportion of patients treated by EI-CXL (6/34, 17.6%) at a mean depth of about 230 μm, whereas it was visible in most of the patients (25/34, 73.5%) at a mean depth of about 300 μm by C-CXL. IVCM images also demonstrated the demarcation zone between the treated and untreated corneal stroma, at a mean depth of (302.0 ± 41.7) μm after C-CXL and of (251.2 ± 28.1) μm after EI-CXL. The depth of corneal demarcation zone detected by IVCM was significantly deeper after C-CXL than after EI-CXL (*P* < 0.001). Representative IVCM images at 3 years after C-CXL and EI-CXL were shown (Fig. 1).

**Fig. 2:**
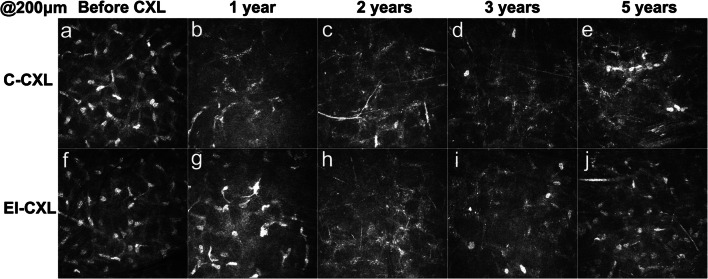
Representative images of IVCM at the depth of 200 μm for C-CXL (**a**–**e**) and EI-CXL (**f**–**j**). Keratocyte repopulation was detectable at all follow-up timepoint after C-CXL and EI-CXL, but the cell density was lower than baseline. Hyperreflective needle-shaped reflection could be observed for both protocols at 2 and 3 years after surgery. At 5 years after surgery, cell repopulation appeared more apparent in EI-CXL group (**j**) than that in C-CXL group (**e**)

Representative IVCM images at 200 μm measured from epithelial surface of each follow-up time were also shown in Fig. 2. Keratocyte population was detectable but decreased at 1, 2, 3, and 5 years after C-CXL (Fig. 2b–f) and EI-CXL (Fig. 2g–j) when compared to the baseline (Fig. 2 a and f). Activated keratocytes with elongated membrane processes and surrounded hyperreflective collagen fibers were detectable in the first postoperative year for both protocols. At 2, 3, and 5 years after surgery, cell repopulation with hyperreflective needle-shaped micro-bands or micro-striate reflections could be observed for both protocols, but the cell repopulation appeared to be more apparent in EI-CXL group at 5 years (Fig. 2j) than that in C-CXL group (Fig. 2e).

### Postoperative complications

After C-CXL, all patients reported obvious pain and discomfort in the first week after surgery, while good toleration to EI-CXL surgery was recorded in 31 patients (31/34, 91.2%). Central corneal epithelial exfoliation (9 mm diameter) after EI-CXL was found in 1 patient (2.9%) with a history of allergic conjunctivitis on day 1 after surgery, but gradually recovered after 7 days. Sterile corneal infiltrate (1/34, 2.9%) and temporary corneal haze (3/34, 8.8%) was recorded after C-CXL, but none (0%) after EI-CXL. No persistent corneal haze, recurrent epithelium erosion, corneal melting, or perforation were noted during the period of follow-up after both CXL protocols. Endothelial cell density and intraocular pressure remained stable after both protocols. Further keratoconus progression was recorded in 2 patients after C-CXL (1 after 3 years and 1 after 4 years) and 3 patients after EI-CXL (2 after 2 years and 1 after 3 years).

## Discussion

In the past decade, although some study demonstrated that iontophoresis-assisted CXL could achieve the same clinical result as standard CXL [21], most evidences have demonstrated inferior results of standard protocol of transepithelial CXL when compared to the conventional epi-off protocol. However, our data indicated that EI-CXL achieved by doubling the iontophoresis cycle displayed approximate long-term(5 years) efficacy with C-CXL in halting the progression of keratoconus in adults. This modified transepithelial protocol might be a good alternative in the management of progressive keratoconus. To our knowledge, our study was the first to demonstrate the long-term effects of transepithelial CXL assisted by iontophoresis for 10 min.

Riboflavin is water soluble and negatively charged at physiological pH. In transepithelial CXL, iontophoresis can be applied effectively to enhance riboflavin penetration. Existing recommendations for iontophoresis in transepithelial CXL utilize 1 mA for 5 min with a 0.1% riboflavin solution. Standard iontophoresis allowed riboflavin imbibition with one-half the concentrations of the C-CXL technique in a rabbit model [8]. Variables in protocols of iontophoresis are able to obtain improved riboflavin penetration. For example, the use of the cationic surfactant benzalkonium chloride has been shown with percutaneous iontophoresis to have a synergistic effect on the transport of anions [6]. An ex vivo study utilized two cycles of iontophoresis each followed by a 5-min soak period to allow time for riboflavin to diffuse more posteriorly, and the stromal riboflavin concentrations were found to be similar to epithelium-off controls [22]. Transepithelial CXL assisted by two continuous cycles of iontophoresis also showed better short-term outcome than that by standard iontophoresis [15]. In our study, no significant differences of the long-term refractive and visual outcome were found between C-CXL and EI-CXL group. The cessation of keratoconus progression with up to 5 years follow-up demonstrated the potential of this modified iontophoretic protocol.

The depth of the acellular zone of anterior corneal stroma after CXL has been correlated with the effectiveness of the CXL treatment [23–25]. The limited penetration depth of riboflavin is one of the critical inadequacies of transepithelial CXL with standard iontophoresis protocol. The penetration depth ranged from 100 to 240 μm in most clinical studies [25–29]. In our previous study [30], the penetration depth of transepithelial CXL using the same iontophoretic device for 5 min was about 133 μm from the corneal surface. The penetration depths in these iontophoretic protocols were all shallower when compared to that in conventional CXL. The huge range of different penetration depth among these reports might be contributed to the variety of iontophoretic devices, riboflavin concentration, and UV radiation parameters used in the CXL surgery. In a series of laboratory investigation, it has been shown that by increasing riboflavin concentration, iontophoresis application times, and allowing short periods of time for riboflavin, which in initially deposited only into the epithelium and anterior stroma, to diffuse deeper into the stroma, concentrations of up to 80% of that achieved with epithelium-off application with a homogeneous distribution throughout the stroma can be achieved [26]. Apparently increased penetration depth could be achieved by repeated iontophoresis. In this study, IVCM showed an average penetration depth of 251 μm in EI-CXL, which achieved an increase of almost 90% of that achieved with iontophoresis using riboflavin 0.1% and 0.1 mA for 5 min in our previous standard iontophoresis (133 μm). The penetration depth in EI-CXL was also far deeper than those achieved with any of the transepithelial CXL protocols using chemical enhancers. With such improved transepithelial penetration depth, EI-CXL achieved approximate results to C-CXL during the follow-up period of 5 years. However, it should be noted that the penetration depth was still more superficial in EI-CXL group than that of C-CXL group.

The statistical analysis showed no differences in the tomographic and refractive parameters between group during the 5-year follow-up (Table 2). Although improvement of *K*_max_, K2, and SE were observed after surgery within both groups, no significant improvement of BCVA was observed in either group of this study. In addition, the refractive data of spherical, cylinder diopter, spherical equivalent, and *K*_max_ showed higher absolute value in the EI-CXL group at 5 years, even though no statistical difference was found. Furthermore, there was a slight increase of corneal stromal cell density and loss of hyperreflective needle-shaped reflection at 5 years after EI-CXL (Fig. 2e), while that in C-CXL group remained almost unchanged (Fig. 2j). This subtle difference of representative IVCM images noted at 5 years after surgery implied that the corneal collagen turnover might happen earlier in EI-CXL group. The earlier turnover of corneal collagen, the more superficial demarcation line in combination with tapering refractive improvement over time after EI-CXL might indicate an inferior efficacy of the EI-CXL protocol when compared with the C-CXL protocol. Further follow-up is needed to determine whether the corneal collagen turnover may induce loss of EI-CXL effect duration with new corneal instability or keratoconus progression in 5 to 10 years after EI-CXL.

The main limitations of our study include the retrospective study design, lack of randomization, and a relatively small sample size that may reduce the power to further interpretation. As a regional eye center in China, our hospital treated a large number of keratoconus patients who lived far away from our hospital. Most of these patients could not guarantee the regular follow-up, increasing the difficulty to conduct a prospective study. In addition, other factors such as age < 14 years, too thin cornea, and *K*_max_ > 60.0 D further cut down the final number of included cases. Furthermore, the treatment protocol was selected and decided by individual patients, depending on their self-estimated tolerance to postoperative pain and acceptance to potential weak effect of epi-on CXL. Besides, patients with more severe keratoconus might prefer C-CXL in order to gain possible better outcome. The lack of randomization inevitably led to selection bias.

In conclusion, transepithelial CXL assisted by enhanced iontophoresis achieved improved stromal penetration depth. Although less than that achieved by conventional epithelium-off protocol, its considerable stromal riboflavin penetration as well as long-term tomographic and refractive improvement, without obvious postoperative complications, demonstrated the potential of EI-CXL to be a better transepithelial protocol. Randomized multicenter clinical trials are needed to further determine the long-term efficacy level.
